# Prevalence of extended-spectrum β-lactamase positive bacteria in radiologically positive urinary tract infection

**DOI:** 10.1186/2193-1801-3-216

**Published:** 2014-05-01

**Authors:** Md Rana Masud, Hafsa Afroz, Md Fakruddin

**Affiliations:** Department of Microbiology, Primeasia University, 12 Kemal Ataturk Avenue, 9 Banani C/A, Dhaka, 1213 Bangladesh; Square Hospitals Limited, 18/F, Bir-Uttam Kazi Nooruzzaman Sarak, West Panthapath, Sher-e Bangla Nagar, Dhaka, 1205 Bangladesh; Institute of Food Science and Technology (IFST), Bangladesh Council of Scientific and Industrial Research (BCSIR), Dhaka, Bangladesh

**Keywords:** UTI, ESBL, Radiological imaging, Antibiotic, Sensitivity

## Abstract

The increase in antibiotic resistance among uropathogens is a global problem. The present study was an effort to assess the current antibiotic resistance pattern and plasmid profiles of some multi drug resistant bacteria isolated from urinary tract infection (UTI). Among 44 clinical samples of radiologically positive UTI, 44 microorganisms belonging to 9 genus were isolated. Of the patients, 24 were female and 20 were male. Highest incidence was found in age group of 30–45 years. Total bacterial count of the urine samples were high in most the patients. *E. coli* and coagulase-negative *Staphylococcus* spp. were most prevalent. Most of the isolates showed higher antibiotic resistance against the antibiotics used. 6 of the 44 isolate was resistant to 10 different types of antibiotics. Of the isolated uropathogens, 40.9% were ESBL positive. 7 of the isolates had no plasmid and 9 isolate had 140 MDa plasmid whereas other isolates pose smaller plasmids of different sizes. Assessment of transfer of antibiotic resistance between different genuses revealed transfer of resistance within genus. Radiological imaging showed strong correlation with microbiological findings of the patients.

## Background

Urinary Tract infection (UTI) is the second most common infectious presentation in community medical practice after the respiratory tract infections (Amin et al. 2009). Worldwide about 150 millions peoples are diagnosed with UTI each year and are classified as uncomplicated or complicated. Complicated UTI include abnormalities of the urinary tract that impede urine flow, the existence of foreign body (e.g. Indwelling catheter, stone) or infection with multi drug resistance pathogen (Beyene and Tsegaye 2011). Urinary tract infection may involve only the lower urinary tract or both the upper and lower tracts. The most common uropathogenisis identified in adult patients with UTIs are the enteric gram negative bacteria. More than 95% UTIs are caused by a single bacterial species. E. coli is the most frequent infecting organisms in acute infection. *Klebsiella*, *Coagulase negative Staphylococci*, *Staphylococci*, *Enterococci*, *Proteous*, *Pseudomonas*, *Enterobacter*, *Candida* are also able to cause UTIs (Forbes et al. 2007). The incidence of urinary tract infection is greatly influenced by age, sex and factors which impair the defense mechanisms that maintain the sterility of the urinary tract (Das et al. 2009). Many predisposing factors have been described for the development of UTI including anatomical, pathological, infective, social and environmental factors (Leigh 1990).

Drug resistance is a natural biological response of microbes involving mutation and survival of the fittest. It becomes a problem only when disease-causing organisms develop the ability to fight off disease curing drugs when these drugs are partially used or used unnecessarily (Meeren et al. 2013). That is how drug resistance is spreading fast mainly due to overuse of antibiotics, incomplete and under use of medications and widespread practice of feeding livestock low levels of antibiotics to promote growth (Islam and Hasan 2012). The interest and need for research on bacterial drug resistance is increasing day by day. This because the transfer of drug resistance among bacterial strains is creating problems in treating bacterial diseases worldwide (Kumar et al. 2006). The increasing resistance pattern to a drug creates pressure to switch to a different more potent drug as prescription. It is also mentionable that just as drug resistance is mainly an acquired property; it can also be lost in course of time (Munshi et al. 1987). That is why it has been observed in many instances that the resistance pattern of some drugs towards a particular pathogen shows rises and downfalls with course of time. Therefore, vigilance is needed in screening the drug resistance pattern of different antibiotics which should be a continuous process.

The aim of the study was to determine antibiotic susceptibility pattern of uropathogens and their plasmid profile and to correlate radiological imaging findings with microorganisms.

## Result

### Age and sex distribution of patients

Of the 44 UTI patient, 24 were female and 20 were male. Age distribution of the patients are given in Table 1.

**Table 1:** **Sample distribution by age category of patient**

Age	Frequency	%
0–15	11	25
15–30	7	15.9
30–45	13	29.5
45–60	7	15.9
60-Above	6	13.6
Total	44	100

### Total count of the urine samples

Total bacterial count in the urine sample is a basis for diagnosis of urinary tract infection. Table 2 shows distribution of patients in term of total bacterial count of urine.

**Table 2:** **Total bacterial count of urine samples**

Total bacterial count (cfu/ml)	No of samples	Percentage (%)
>10^3^	3	6.81
>10^4^	5	11.36
>10^5^	14	31.81
>10^6^	13	29.54
>10^7^	6	13.63
>10^8^	3	6.81
Total	44	100

### Identification of uropathogens

44 microorganisms has been isolated from the urine samples and identified to genus level. 9 different genus was identified. *E. coli* and coagulase-negative *Staphylococcus* spp. were most prevalent in the samples (Table 3).

**Table 3:** **Bacteria isolation from urine sample**

Name of organisms	Frequency	Percentage (%)
*E. coli*	13	29.54
*Coagulase negative Staphylococcus spp.*	9	20.45
*Enterococcus spp*	3	6.81
*Klebsiella spp*	4	9.09
*Morganella morganili*	3	6.81
*Acinetobacter spp.*	2	4.54
*Citrobacter spp.*	3	6.81
*Pseudomonas* spp.	5	11.36
*Proteous spp.*	2	4.54

### Antibiotic susceptibility of isolated uropathogens

The majority of the isolates were resistant to the antibiotics used. Highest resistance rate was found against chloramphenicol, ampicillin and penicillin. Cefixime and cotrimoxazole showed to be better antibiotic against the uropathogens (Table 4).

**Table 4:** **Antibiotic resistance pattern of the isolates**

Name of antibiotic	Percentage (%)	Name of antibiotic	Percentage (%)
Amoxicillin (AMX)	77.27	Ceftriaxone (CFT)	56.81
AmoxyClav (AMV)	52.27	Cefuroxime (CFU)	52.27
Cefepime (CEF)	59.09	Ciprofloxacin (CIP)	70.45
Cefixime (CFX)	50	Gentamycin (GEN)	79.54
Chloramphenicol (CHL)	81.81	Cefoxitin (GEN)	43.18
Cotrimoxazole (COT)	47.72	Erythromycin (CXT)	75
Amikacin (AMK)	61.36	Penicillin (PEN)	84.09
Ampicillin (AMP)	86.36	Nitrofurantoin (NIT)	63.63

Distribution of microorganisms according to the number of antibiotics they are resistant to shows that most of the isolates were resistant to at least 3 antibiotic (Table 5).

**Table 5:** **Distribution of antibiotic resistance**

No of antibiotic	No of isolate resistant (%)
10 Types of Antibiotic	6 (13.63)
8 Types of Antibiotic	9 (20.45)
6 Types of Antibiotic	11 (25)
4 Types of Antibiotic	9 (20.45)
2 Types of Antibiotic	4 (9.09)
1 Types of Antibiotic	2 (4.54)
0 Types of Antibiotic	3 (6.81)

### Prevalence of ESBL-producing organisms

Identified isolates were further tested for the production of ESBL. 18 (40.9%) of the total 44 uropathogens identified were positive for ESBL. Of 13 *E. coli*, 7 were ESBL positive; of 9 coagulase-negative *Staphylococcus* spp., 3 were ESBL positive. None of the *Proteus* spp. and *Morganella morganii* isolated were ESBL positive. ESBL positive isolate was found in all other genus isolated (Table 6).

**Table 6:** **ESBL positive isolates from UTI**

Name of organisms	Number of isolate	ESBL positive	ESBL negative
*E. coli*	13	7	6
*Coagulase negative Staphylococcus spp.*	9	3	6
*Enterococcus spp*	3	2	1
*Klebsiella spp*	4	1	3
*Morganella morganii*	3	0	3
*Acinetobacter spp.*	2	2	0
*Klebsiella spp.*	3	2	1
*Pseudomonas* spp.	5	1	4
*Proteous spp.*	2	0	2
Total	44	18 (40.9%)	26 (59.1%)

### Plasmid profiling

Among the 44 UTI isolates, 7 showed no plasmid bands, 9 showed only a single large plasmid band around 140 MDa marker. The rest of the isolates showed multiple plasmid bands ranging from very large to less than 2 MDa. Distribution of isolates according to harboring plasmid is given in Table 7.

**Table 7:** **Distribution of isolates according to plasmid**

Plasmid size	No of isolate (%)
No plasmid	7 (15.9)
~140 MDa	9 (20.5)
~2.7 MDa	25 (56.8)
<2.1 MDa	14 (31.8)

### Transfer of antibiotic resistance by conjugation

Transfer of antibiotic resistance between 3 pairs were assessed- *E. coli* isolate-2 & *E. coli* isolate-5; *E. coli* isolate-2 & *Klebsiella* spp. isolate-1; *E. coli* isolate-2 & *Staphylococcus* spp. isolate-3. Transfer of resistance was observed in each of the 3 pairs (Table 8).

**Table 8:** **Transfer of antibiotic resistance between isolates of different genus**

Mating pair	Resistance pattern											
	Before incubation						After incubation					
	AMP	CHL	CFX	CIP	CFT	GEN	AMP	CHL	CFX	CIP	CFT	GEN
*E. coli + E. coli*	R	R	S	***S***	***R***	***S***	R	R	S	***R***	***R***	***R***
	R	R	S	***R***	***S***	***R***	R	R	S	***R***	***R***	***R***
*E. coli + Klebsiella*	***R***	R	***S***	***S***	***R***	S	***R***	R	***R***	***R***	***R***	S
	***S***	R	***R***	***R***	***S***	S	***R***	R	***R***	***R***	***R***	S
*E. coli + Staphylococcus*	***R***	R	S	S	***R***	R	***R***	R	S	S	***R***	R
	***S***	R	S	S	***S***	R	***R***	R	S	S	***R***	R

(AMP = Ampicillin; CHL = Chloramphenicol; CFX = Cefixime; CIP = Ciprofloxacin; CFT = Ceftriaxone; GEN = Gentamycin; S = Sensitive; R = Resistant; Transfer of antibiotic resistance has been highlighted italic bold).

### Correlation of radiological imaging with microorganisms

Radiological (RGU-MCU & USG) findings of the patients were analyzed according to total bacterial count of the urine of the patients. Patients with imaging findings and symptoms of UTI has the following total bacterial count as presented in Table 9.

**Table 9:** **Correlation of radiological findings with total bacterial count**

Total bacterial count (cfu/ml)	RGU-MCU	USG
>10^3^	+	+
>10^4^	+	+
>10^5^	+	+
>10^6^	+	+
>10^7^	+	+
>10^8^	+	+

## Discussion

Urinary tract infections are common conditions worldwide and the pattern of antimicrobial resistance varies in different regions. The interest and need for research on bacterial drug resistance is increasing day by day. The reason is, the transfer of drug resistance among bacterial strains is creating problems in treating bacterial diseases worldwide. The increasing resistance pattern to a drug creates pressure to switch to a different more potent drug as prescription. It is also mentionable that just as drug resistance is mainly an acquired property; it can also be lost in course of time. That is why it has been observed in many instances that the resistance pattern of some drugs towards a particular pathogen shows rises and downfalls with course of time. Therefore, vigilance is needed in screening the drug resistance pattern of different antibiotics which should be a continuous process.

A total of 44 samples were analyzed in this study, which showed positive UTI in radiological imaging procedure. The urine samples of radiologically positive patients were collected and analyzed by standard microbiological procedure. Of the 44 patients, 24 (54.54%) were female and 20 (45.45%) were male. UTI was more prevalent in age group 30–45 years. A previous study by Ahmed et al. ( 2011) showed that UTI in Bangladesh is more prevalent in females and in age 15–40 years. Prevalence of UTI in infants and young (0–15 age group) is also high (25%) which is alarming.

Coulthard et al. ( 2010) recommended threshold value of total bacterial count of urine for diagnosis of UTI should be ≥10^6^ cfu/ml but in present study with radiologically positive UTI patients, total bacterial count of urine ranged from >10^3^- > 10^8^ cfu/ml. 50% (n = 22) of the patients showed >10^6^ cfu/ml total bacterial count. 44 bacterial isolate was identified to genus level on the basis of biochemical characters. Bacteria belonging to 9 different genus had been isolated. Among the causative agents- *E. coli* was found to be most prevalent (29.54%), followed by *coagulase-negative Staphylococcus spp.* (20.45%). Other genus identified were *Enterococcus* spp. (6.81%), *Klebsiella* spp. (9.09%), *Morganella morganii* (6.81%), *Acinetobacter* spp. (4.54%), *Citrobacter* spp. (6.81%), *Pseudomonas* spp. (11.36%) and *Proteus* spp. (4.54%). This study in accordance with many previous studies conducted in Bangladesh those also showed *E. coli* to be the most prevalent pathogen associated with UTI (Islam and Hasan 2012; Khan et al. 2013; Akter et al. 2013; Shilpi et al. 2012; Rahman et al. 2009).

Antibiotic resistance in the urinary isolates were found to be high against the 16 antibiotic used in this study. The isolates showed higher resistance to chloramphenicol (81.81%), ampicillin (86.36%), penicillin (84.09%), and gentamycin (79.54%). Lower resistance was found against cefixime (50%), cotrimoxazole (47.72%), cefoxitin (43.18%). Increasing pattern of resistance of urinary tract pathogens against common antibiotics in Bangladesh have been reported by other researchers (Shilpi et al. 2013; Noor et al. 2013; Gomes et al. 2011; Jhora and Paul 2011; Jhora et al. 2011). 13.63% isolates were resistant to 10 (out of 16) antibiotics, 20.45% were resistant to 8 antibitotics, 25% were resistant to 6 antibiotic, 20.45% were resistant to 4 antibiotic. Only 6.81% isolates were sensitive to all the antibiotics used. 40.9% (n = 18) of the isolates were found to be ESBL positive. Of the ESBL positive isolates, *E. coli* was the prevalent organism (Table 6). A recent study in Bangladesh showed 43% (86 out of 200 isolate) ESBL positive bacteria in urinary tract infection where *E. coli* was most prevalent (Khan et al. 2013). A considerable increase in resistance was observed against amoxicillin, ampicillin, gentamicin, ciprofloxacin, amikacin and ceftriaxone. However, cefixime, cotrimoxazole, nitrofurantoin and cefoxitin resistance were more or less in accordance with the previous pattern. An ovarall observation is clearly indicative of continuous rise in drug resistance of urianry pathogens in Bangladesh.

Antibiotic resistant genes usually reside in plasmid and transfer of antibiotic resistance are mostly plasmid mediated (Munshi et al. 1987). Plasmid profile of the isolates showed all isolate except seven pose plasmids of different sizes. 9 isolate had single plasmid of around 140 MDa size. This large plasmid in has been reported in many multi-drug resistant bacteria worldwide. The rest of the isolates showed multiple plasmid bands ranging from very large to less than 2 MDa (Table 7). Transfer of antibiotic resistance between urinary tract isolates were assessed and it was found that resistance can be transferred both intra-genus and inter-genus (Table 8). Most alarming finding is transfer of antibiotic resistance from *E. coli*, a gram negative organism to *Staphylococcus* spp., which is a gram-positive organism. This finding is supported by the study of Courvalin ( 1994) who showed transfer of antibiotic resistance genes between Gram-negative and Gram-positive bacteria.

From this study Radiological imaging procedure seemed to have comparable sensitivity than standard microbiological procedures for detecting old/chronic UTI cases (Table 9). Yoon et al. ( 2011) also reported that there is no significant differences in the image findings of UTI with findings for causative microorganisms. However, it can be concluded that radiological study should be applied along with microbiological analysis for proper choice of drug and better treatment.

## Conclusion

It is quite alarming to note that almost all of the isolates included in this study were found resistant to multiple drugs (four or more antibiotics). Antibiotic resistance has been emerged as a major problem in the management of hospitalized patients as well as those with chronic conditions and adds considerably to health care cost. It has been considered a threat to the public health problem worldwide. This important issue is to be addressed by the policy makers to formulate a strict antibiotics prescription policy in our country, which would aware the practitioners and care giver to make a prudent use antibiotics.

## Materials and methods

### Ethical statement

Ethical clearance was obtained prior to data collection and enrollment of patients from the Ethical Review committee of Square Hospital Ltd., Dhaka, Bangladesh and patients were enrolled after written informed consent was obtained.

### Study subjects

In total, 44 imaging positive patients who had clinical symptoms of UTI were identified and included in the present study at Square Hospital Ltd. Dhaka Bangladesh, a tertiary care hospital. There were 44 patients with an age range of 0-above 60 years.

### Urine sample collection

According to Sonnenwirth and Jerett ( 1980), the urine sample was collected in the pre-sterile dried test tube. Early morning urine is preferred. Patient voided and gave the midstream urine in sterile container, attendant following precautions in order to avoid contamination. Guideline s for proper specimen collection was given to all patients on a printed card Urine samples were processed immediately, but in case of delay they were refrigerated at 4°C until processing.

### Sample preparation

30 milliliters of urine samples was taken in a clean sterile centrifuge tube and centrifuged at 3000 rpm for 10 minutes. The supernatant was discarded aseptically and the sediment in the centrifuge tube was homogenized in 100 μl of supernatant. The homogenized suspension was then used for total bacterial count and identification of uropathogens.

### Enumeration of microorganisms

The bacteria count was performed standard method. The microbiological condition safety and hygiene were then assayed using the methods recommended by ICNSF. Equal or more than 10^4^ CFU/ml of single potential pathogen or for each of two potential pathogens interpreted as positive UTI.

### Isolation and identification of uropathogens

Uropathogens were identified through culture, microscopy, and biochemical tests. HighChrome chromogenic agar media, MacConkey agar, blood agar, chocolate agar, and Oxoid clarity agar media (Oxoid Ltd., Hampshire, UK) were inoculated with 100 μL of prepared urine samples, and incubated at 37°C for 24 hours. Bacterial identification was done by phenotypic examination of the culture, looking for typical characteristics, and by Gram staining, and a series of standard biochemical tests were also performed to identify the bacteria of interest (Cappuccino and Sherman 1996).

### Antibiotic susceptibility testing

The agar disc diffusion assay was used to determine the antimicrobial susceptibilities of uropathogens. The discs used in this study included Amoxicillin 30 μg, AmoxyClav 30 μg, Cefepime (4th) 30 μg, Cefixime (3rd) 5 μg, Chloramphenicol 30 μg, Cotrimazol 25 μg, Amikacin 10 μg, Ampicilin 30 μg, Ceftriaxone (3rd) 30 μg, Cefuroxime (2nd) 30 μg, Ciprofloxacin 5 μg, Gentamycin 10 μg, Cefoxitin 30 μg, Erytromycin 15 μg, Penicillin 10 μg, Nitrofurantoin 300 μg. The protocol for antibiotic susceptibility testing has been described previously (Bauer et al. 1966). The diameters of the zones of inhibition for individual antimicrobial agents were translated into susceptible, intermediate, and resistant categories according to National Committee for Clinical Laboratory Standards criteria ((CLSI) 2011).

### Detection of ESBL by the double disc diffusion method

Positive (*K. pneumoniae* ATCC 700603) and negative (*E. coli* ATCC 25922) control strains were inoculated onto Muller-Hinton agar (HiMedia Laboratories Pvt. Ltd., Mumbai, India). An amoxicillin-clavulanic acid (20 mg & 10 mg) disc was placed on the center of the plate. Ceftazidime (30 mg), ceftriaxone (30 mg), cefotaxime (30 mg), and aztreonam (30 mg) discs were placed peripherally away from the amoxicillin clavulanic acid disc. After 24 hours of incubation at 37°C, band formation between the amoxicillin clavulanic acid disc and any other disc was considered ESBL positive. The ESBL positive strains were further subjected to phenotypic confirmatory tests using sensitivity discs, which contained third-generation cephalosporins both with and without clavulanic acid. The discs used included cefotaxime (30 mg), cefotaxime & clavulanic acid (30 mg & 10 mg), ceftazidime (30 mg), ceftazidime (30 mg & 10 mg), aztreonam (10 mg), and aztreonam & clavulanic acid (30 mg & 10 mg). The differences in the zone of inhibition caused by the cephalosporins alone and when combined with clavulanic acid were recorded and if the difference was 5 mm or more, the strains were confirmed as ESBL-producing strains (Kumar et al. 2006).

### Plasmid DNA analysis

Plasmid DNA was prepared by SDS/alkali lysis method and electophoresed using 0.8% agarose in TBE buffer at 100 V for 30 minutes, in a horizontal electrophoresis system. Gels were stained with 0.1 μg/ml of ethidium bromide for 20 minutes. The gels were viewed on an ultraviolet transilluminator and photographed by using a Polaroid camera. Plasmids of *E. coli* PDK9 were used as plasmid molecular weight markers (Gomes et al. 2011).

### Analysis of transmission of antibiotic resistances by conjugation

Transfer of antibiotic resistance within genus was studied using conjugation. On the basis of antibiotic resistance profile of the isolates, 3 pairs were selected- *E. coli* isolate-2 & *E. coli* isolate-5; *E. coli* isolate-2 & *Klebsiella* spp. isolate-1; *E. coli* isolate-2 & *Staphylococcus* spp. isolate-3. Overnight cultures were grown to an A_540_ of 0.1 (approximately 10^8^ cells/mL). Five milliliters of each participating bacterial culture was mixed (1:1) in a test tube containing sterile Luria Bertani (LB) broth and incubated without shaking for 24 h at 36 ± 1°C. The transconjugants were selected on the LB agar plates supplemented with following antibiotics, viz. ampicillin, chloramphenicol, cefixime, ciprofloxacin, ceftriaxone and gentamycin (Vaidya 2011).

### Radiological imaging

Imaging studies are tests performed with a variety of techniques that produce pictures of the inside of a patient’s body. Imaging tests are performed using sound waves, radioactive particles, magnetic fields, or x rays that are detected and converted into images after passing through body tissues. In the study, two types of special techniques were used. (i) Retrograde urethrography (RGU) with Micturating Cystourethrography (MCU) (ii) Ultrasonography (USG). Imaging study is appropriate in humans after the first onset of UTIs. USG for the detection of congenital abnormalities, obstruction, wall thickening of urinary bladder, Vesicoureteric reflux (VUR) and Cystitis. RGU with MCU identified of VUR, Stricture of urethra, Cystitis and incomplete voiding of urinary bladder (Yoon et al. 2011).
